# Latent Profiles of Parental Burnout During COVID-19: The Role of Child-Related Perceptions

**DOI:** 10.3389/fpsyg.2021.682642

**Published:** 2021-09-03

**Authors:** Katja Upadyaya, Katariina Salmela-Aro

**Affiliations:** Faculty of Educational Sciences, University of Helsinki, Helsinki, Finland

**Keywords:** parental burnout, strengths and difficulties, latent profile analysis, growth mindset, COVID-19

## Abstract

The present study examined latent profiles of parental burnout dimensions (e.g., exhaustion in parental role, contrast with previous parental self, feelings of being fed up, and emotional distancing, measured with a shortened version of the parental burnout assessment scale) among Finnish parents of sixth and eighth grade children. In addition, the role of children’s strengths and difficulties (e.g., prosocial skills, hyperactivity, somatic problems, conduct problems, and peer problems) and parents’ growth mindset in predicting membership in the latent parental burnout profiles was examined. The participants were 1,314 parents (80% mothers) from the Helsinki Metropolitan area who filled in a questionnaire concerning their parenting burnout and child-related perceptions during the fall 2020. The results were analyzed using latent profile analysis (LPA) and three-step procedure. Three latent profiles of parental burnout were identified as: low parental burnout (85.7% of the parents), high parental burnout (8%), and emotionally distanced (6.3%) profiles. Parents who reported their children having some challenges (e.g., hyperactivity, somatic problems, conduct problems, and peer problems) more often belonged to the high burnout or emotionally distanced profiles rather than to the low parental burnout profile. Parents whose children had high prosocial skills and who employed growth mindset more often belonged to the low parental burnout rather than to the distanced profile.

## Introduction

For many parents, parenting is a highly rewarding experience with multiple positive consequences, such as increases in the meaning of life, happiness, and wellbeing (Nelson et al., 2013). However, parenting can also be taxing and taking care of children may involve both acute (e.g., conflicts) and chronic stressors (e.g., behavioral problems and health issues; Mikolajczak et al., 2019). Especially, when parents’ resources do not meet the parenting demands, and the difficulties to deal with the existing stressors are overwhelming, parents may be at risk for parental burnout (Mikolajczak et al., 2019, 2020). A completely new and unexpected environmental stressor occurred in 2020, when the COVID-19 pandemic spread across the world. The pandemic resulted in lockdowns and quarantines across countries, causing severe turmoil in many families’ lives. Due to the COVID-19 outbreak, concerns and fears about the virus increased which may have led to altered levels of stress, anxiety, and parental burnout among many families (Prikhidko et al., 2020). As the schools and workplaces were closing, many parents had to supervise their children’s schooling at home while simultaneously managing their own work remotely. In Finland, most schools were closed nearly 2months during the initial phase of COVID-19 in spring 2020. However, they were again opened during the fall 2020, the time when the current data were collected. At the same time, lockdowns caused severe financial strain in many families’ lives, and while parents needed to multitask and balance with their work and family duties, challenges related to these unexpected changes in work and family life may have resulted in altered parental burnout symptoms (see also Griffith, 2020).

The field studying parental burnout is relatively new; however, it is clear that parental burnout is a serious condition which would deserve heightened attention (Mikolajczak et al., 2019). Parental burnout can be highly damaging, and can manifest as suicidal and escape ideations (Mikolajczak et al., 2019), and as child neglect or abuse (Mikolajczak et al., 2018). Another concern parental burnout rises is its prevalence, as a study examining parents from 42 countries showed that parents across the world, especially in individualistic Western cultures, such as Finland, United Kingdom, Belgium, and United States, report symptoms of parental burnout (Roskam et al., 2021). Thus, it is of great importance to examine in more detail what percentage of parents experiences high or altered levels of parental burnout, and what factors precede it. However, even several studies have examined the different subdimensions of parental burnout, their antecedents and outcomes (Roskam et al., 2018), person-oriented research examining latent profiles of parental burnout is still sparse (see Hansotte et al., 2021; Lebert-Charron et al., 2021 for exceptions). It is possible that parents report different levels of burnout symptoms and that distinct latent homogeneous profiles can be identified reflecting high, average, and low levels of parental burnout. These profiles can be examined using person-oriented research, such as latent profile analysis (LPA; Muthén and Muthén, 2021). Consequently, the present study was among the first examining latent profiles of parental burnout (e.g., exhaustion in parental role, contrast with previous parental self, feelings of being fed up, and emotional distancing) by means of LPA during the pandemic. In addition, the role of parental perceptions (e.g., strengths and difficulties and growth mindset) in predicting membership in the latent profiles was examined.

### Parental Burnout

Parental burnout develops as a prolonged response to overwhelming parental stress, when parents’ own resources do not meet the parenting demands (Mikolajczak and Roskam, 2018). Parental burnout has been distinguished from job burnout, as one can simultaneously be drained by one’s job and not by parenting, and *vice versa* (Mikolajczak et al., 2019), and even parental burnout literature shares some similar aspects (e.g., exhaustion) with job burnout literature, and parental burnout is mildly correlated with job burnout, it loads to separate factors from job burnout (Roskam et al., 2017, 2018). Parental burnout was initially researched using similar constructs to job burnout (e.g., exhaustion, depersonalization, and inefficacy) using the parental burnout inventory (PBI; Roskam et al., 2017); however, further research among burned out parents indicated the existence of four separate dimensions specific to parental burnout, each of which describes different aspects of parents’ experiences. These four dimensions are measured with parental burnout assessment (PBA; Roskam et al., 2018). The most important dimension of parental burnout is *exhaustion*, which describes parents’ feelings of tiredness in parental role, so that it reaches the level of exhaustion (Roskam et al., 2018). The second dimensions are called as *contrast with previous parental self*, which describes parents’ feelings of not being able to be as good parent as before (Mikolajczak et al., 2019). As a diagnostic criterion of parental burnout, contrast with previous self-distinguishes exhausted parents from permanently dismissive ones (Roskam et al., 2018). The third dimension, described as parents’ *feelings of being fed up*, characterizes parents’ loss of pleasure and feelings of fulfillment in parental role (Roskam et al., 2018). The fourth dimension is characterized as parents’ *emotional distancing from one’s children*, when parents are so exhausted they disengage emotionally rather than physically from their children (which is often not possible), and provide only the necessary practical care, such as taking care of everyday tasks, but become emotionally less involved and responsive to their children (Roskam et al., 2018). It can be further assumed that different crisis situations alter burnout symptoms among parents. The present study examined parental burnout profiles during the COVID-19 pandemic.

It is also possible that experiences of parental burnout are not similar for all parents. For example, some parents may experience generalized exhaustion in parental role, whereas other parents feel increased emotional distance from their children. These differences can be captured using person-oriented methods, such as LPA, which was employed in the present study. The structure of parental burnout dimensions has been examined to some extent (Roskam et al., 2018; Aunola et al., 2020), however, so far two studies have previously sought to examine parental burnout profiles using LPA or cluster analysis as a method (Hansotte et al., 2021; Lebert-Charron et al., 2021). In their study, using the PBA, Lebert-Charron et al. (2021) examined over one thousand French mothers and were able to identify five clusters on the basis of parental burnout symptoms. The largest cluster (49% of the mothers) was called as “absence of parental burnout,” characterized by very low scores on all burnout dimensions. The second largest group (18%), “middle manifestations of parental burnout” cluster, described low levels of exhaustion and saturation, and very low levels of contrast and emotional distancing. The third (11%) cluster described altered emotional distancing, and the fourth (12%) and fifth (10%) clusters described high and very high manifestations of parental burnout. Group differences were found between the clusters concerning affective variables, such as anxiety, burden, and depressive symptoms, which were altered in clusters reflecting higher levels of parental burnout (Lebert-Charron et al., 2021). Similarly, using the earlier measure of PBI and an online survey, Hansotte et al. (2021) were able to identify five latent parental burnout profiles: not in parental burnout (59%), inefficient (9%), at risk of parental burnout (20%), emotionally exhausted and distant (8%), and burned out (3%) profiles. The results further showed that profiles with high levels of exhaustion and emotional distancing were associated with higher levels of neglect and violence (Hansotte et al., 2021). The present study continues this line of person-oriented research on parental burnout, using LPA as a method. The advantage of LPA over traditional cluster analysis used in the previous study is that it is model-based and provides fit indices for different latent profile solutions, which can then be compared in order to determine the final number of profiles. Moreover, the present study examines parental burnout profiles both among mothers and fathers.

### Parental Burnout and Parental Perceptions Concerning Their Children

Research on parental burnout has often focused on examining various factors that may make parents vulnerable for burning out in parenting (Mikolajczak et al., 2019). One major factor contributing to parental burnout is parents’ concern about their children, such as worries about behavioral or health problems and educational difficulties (Griffith, 2020). Parents of children with chronic illnesses or special needs score higher for burnout than parents of control group (Lindström et al., 2011; Gérain and Zech, 2018). Similarly, parents’ concerns about their children’s behavioral and emotional problems may increase symptoms of parental burnout. The COVID-19 pandemic might have increased also children’s concern about the virus, and as new social distancing recommendations took place and children could spend less time with their friends, which might have shown as problems in their behavior and emotional state. This might have increased parents’ concerns about their children and further manifest in their perceptions of their children. As a result, parents’ perceptions of their children’s strengths and difficulties, such as prosocial skills, hyperactivity, somatic problems, conduct problems, and peer problems (e.g., loneliness), might have amplified during the pandemic’s school closures when parents spent more time with their children at home.

In addition to strengths and difficulties, parents’ perceptions of growth mindset (Dweck and Yeager, 2019) may affect their parenting stress. Growth mindset refers to a belief that one’s capacities are not fixed but can be developed over time, whereas fixed mindset refers to a belief that capacities cannot be shaped or developed (Dweck and Yeager, 2019). Such beliefs can shape one’s experiences and show in their attitudes toward others or learning (Dweck and Yeager, 2019). Some parents emphasize more the possibilities of growth and malleability of abilities (e.g., growth mindset; Dweck and Yeager, 2019), which might have helped parents to feel less stressed about their children’s skill development while homeschooling their children (see also Mosanya, 2020), and result as lesser parental burnout. Contrary to fixed mindset, parents who employ growth mindset believe that children’s capacities are not fixed but can develop over time (Dweck and Yeager, 2019). Parents growth mindset is often associated with parents’ behavior, children’s mindsets (Dweck and Yeager, 2019), and skill development (Andersen and Nielsen, 2016). Recent results have shown, that growth mindset shows as reduced academic stress among university students, and may enhance one’s resilience during times of crisis, such as COVID-19 (Mosanya, 2020). Similarly, parents’ growth mindset might act as a resilience factor and protect parents against severe stress (e.g., parental burnout). However, no previous studies have examined the associations between parents’ perceptions of their children’s strength and difficulties, their own growth mindset, and parental burnout. The present study is the first to examine these associations.

Previous studies have also shown that mothers often experience higher parental burnout than fathers (Aunola et al., 2020; Roskam and Mikolajczak, 2020). Mothers are often more involved in taking care of children than fathers (Mikolajczak et al., 2018) which makes them prone to parental stress and burnout (Aunola et al., 2020). Parents’ educational level is often unassociated with parental burnout (Mikolajczak et al., 2018; Roskam et al., 2018; Aunola et al., 2020); however, less is known about the extent to which parents’ educational level is associated with parenting burnout profiles. The present study examined the role of parents’ gender and educational level in predicting their membership in parental burnout profiles during the pandemic.

### Aims

The following research questions were examined in the present study:

1. What kind of distinct latent profiles (e.g., groups of homogeneous subjects) can be identified according to parental burnout symptoms (e.g., exhaustion in parenting, contrast with previous parental self, feelings of being fed up, and emotional distancing) among Finnish parents during fall 2020?
2. To what extent parents’ perceptions of their children’s strengths and difficulties (e.g., prosocial skills, hyperactivity, somatic problems, conduct problems, and peer problems) predict parents’ belonging to different parental burnout profiles?
3. To what extent parents’ growth mindset predicts them belonging to different parental burnout profiles?
4. To what extent parents’ gender and educational level predict them belonging to different parental burnout profiles?

## Materials and Methods

### Participants

The participants of the present study came from the longitudinal growing mind study. During the fall 2020, during the COVID-19 pandemic, 1,314 parents (80% mothers, 19% fathers, and 1% else) from the Helsinki Metropolitan area filled in a questionnaire concerning their parental burnout and perceptions concerning their sixth and eighth grade children. Finnish children typically start their sixth and eighth grades when they are 12 and 14years old. The parents’ educational level was as follows: elementary education (2%), high school degree (3%), vocational degree (13%), double degree (4%), polytechnic degree (22%), bachelor’s degree (6%), master’s degree (40%), doctoral degree (7%), and other (3%). Most families (77%) had two parents, and 5% consisted of single parents. Altogether 22% of the parents were divorced, and some (8%) lived in blended families.

### Measures

*Parental burnout* was examined with a shortened version of the PBA scale (Roskam et al., 2018). The scale consisted of eight items (e.g., two items concerning each subdimension) measuring parents’ exhaustion in parenting (e.g., “I feel completely run down by my role as a parent”), contrast with previous parental self (e.g., “I tell myself that I’m no longer the parent I used to be”), feelings of being fed up (e.g., “I cannot stand my role as father/mother any more”), and emotional distancing from one’s children (e.g., “I do what I’m supposed to do for my child(ren), but nothing more”). Parents’ answered to each item using a 7-point Likert scale (1 = completely disagree; 7 = completely agree). Sum scores were constructed separately for each parental burnout dimension. The Cronbach’s alpha reliabilities for exhaustion, contrast, feelings of fed up, and distancing were 0.79, 0.70, 0.51, and 0.51, respectively, indicating a moderate to high reliability of the variables (e.g., values between 0.50 and 0.70 considered as moderate reliability; Perry et al., 2004).

*Strengths and difficulties* questionnaire (SDQ, Goodman, 2001) was used to measure parents’ perceptions concerning their children, which is a widely used short tool for emotional and behavioral screening. The questionnaire maps five different dimensions of children’s strengths and weaknesses: prosocial skills (e.g., *“Considers other people’s feelings.”*), hyperactivity (e.g., “*Restless, over-active, unable to be quiet and still for a long time.”*), somatic problems (e.g., *“Often complains about headaches, stomach ache or nausea.”*), conduct problems (e.g., *“Generously shares his/her items with other children.”*), and peer problems (e.g., *“He/she is picked on or bullied by other children.”*). Each subdimension was measured with five items, and parents responded to them with a 3-point scale (1 = false; 3 = entirely true). Sum scores were constructed for each dimension. The Cronbach’s alpha reliabilities for prosocial skills, hyperactivity, somatic problems, conduct problems, and peer problems were 0.72, 0.78, 0.72, 0.56, and 0.60.

*Parents’ growth mindset* was examined with a four questions (Dweck, 2006; e.g., *“A person can learn new things, but he/she cannot change his/her intelligence.”*). Parents answered to the questions with a 6-point Likert scale (1 = completely agree; 6 = completely disagree). The Cronbach’s *α* for the sum score was 0.92.

*Parents’ gender* was coded 1 = mother; 2 = father.

*Parents’ educational level* was coded 1 = basic education; 2 = secondary education; and 3 = tertiary education.

### Analysis Strategy

To be able to identify the homogeneous latent groups of parents with different levels of exhaustion in parental role, contrast with previous parental self, feelings of being fed up, and emotional distancing, the results were analyzed by means of LPA (Muthén and Muthén, 2021), which is a type of finite mixture analysis that assesses heterogeneity through the identification of homogeneous subgroups (i.e., latent profiles) of participants with similar indicator means (e.g., parental burnout dimensions) within the latent profiles. The advantage of LPA over traditional cluster analysis is that it is model-based and provides fit indices for different latent profile solutions, which can then be compared in order to determine the best fitting final solution.

No control variables were used in defining the latent profiles. The latent profile analyses were carried out in two phases. As we were interested in examining what kind of naturally occurring latent profiles of parental burnout indicators could be identified, latent profile analyses for different latent groups were carried out first, and the fit indices and class frequencies were compared. The variances were estimated equal between the classes. The estimation was performed step by step, starting from one-class solution to estimate the parameters for 2, 3, …, *k*-class solutions. The solution that best fitted the data in accordance with the indicators and that was also deemed reasonable in terms of interpretation was chosen as the final latent profile model. Second, in order to identify the possible antecedents of parental burnout profiles, parents’ perceptions of their children’s strengths and difficulties (prosocial skills, hyperactivity, somatic problems, conduct problems, and peer problems), mindset, and parents’ gender and educational level were added into the final model as covariates using the three-step procedure (Asparouhov and Muthén, 2014). In the three-step procedure, after determining the number of latent profiles (step 1, as described above), the profile probabilities were saved in a new data set with the covariates (step 2), and using the new data set, the role of the antecedents was examined further (step 3; see Asparouhov and Muthén, 2014 for further details of the analyses). The benefit of the three-step procedure is that the forming of the latent profiles is free from the effect of the covariates. Each covariate was added in the model separately (see Table 1 for means, variances, and correlations).

**Table 1:** Pearson correlation coefficients, means, and variances for all the examined variables.

S. No.		1	2	3	4	5	6	7	8	9	10	11	12
1.	Parental exhaustion												
2.	Contrast	0.49^***^											
3.	Feelings of fed up	0.57^***^	0.46^***^										
4.	Emotional Distancing	0.37^***^	0.42^***^	0.38^***^									
5.	SDQ prosocial	−0.22^***^	−0.18^***^	−0.20^***^	−0.18^***^								
6.	SDQ hyperactive	0.31^***^	0.24^***^	0.29^***^	0.18^***^	−0.30^***^							
7.	SDQ somatic	0.27^***^	0.22^***^	0.22^***^	0.12^***^	−0.16^***^	0.28^***^						
8.	SDQ conduct problems	0.32^***^	0.26^***^	0.29^***^	0.15^***^	−0.45^***^	0.51^***^	0.31^***^					
9.	SDQ peer problems	0.21^***^	0.21^***^	0.18^***^	0.15^***^	−0.27^***^	0.18^***^	0.36^***^	0.19^***^				
10.	Growth mindset	−0.09^***^	−0.07^**^	−0.09^**^	−0.14^***^	0.10^***^	−0.10^***^	−0.01	0.12^***^	−0.08			
11.	Gender	−0.03	0.02	−0.03	0.11^***^	−0.04	−0.04	−0.03	−0.05	0.00	−0.06^*^		
12.	Educational level	−0.01	−0.03	−0.01	−0.05	0.05	−0.09^***^	0.00	−0.01	−0.04	0.05	0.03	
*M*		1.94	1.75	1.30	1.39	2.44	1.44	1.25	1.28	1.36	4.45	1.19	2.75
*Var*		1.42	1.21	0.42	0.58	0.16	0.18	0.11	0.08	0.10	1.46	0.16	0.22

*** *p*<0.001;

** *p*<0.01;

* *p*<0.05.

All the analyses for the LPAs were performed with the Mplus statistical package (version 8; Muthén and Muthén, 2021). Missing data were deleted listwise, which was the default for this type of analysis (Muthén and Muthén, 2021). There were 4–6% random missingness in the examined variables. The model parameters were estimated by means of maximum likelihood robust (MLR) estimator, which is robust to the non-normality of the observed variables. Maximum likelihood robust produces standard errors and a chi-square test statistic for missing data with non-normal outcomes by means of a sandwich estimator and the Yuan-Bentler T2 test statistic (Muthén and Muthén, 2021).

## Results

The purpose of the mixture analyses was to find out whether distinct latent profiles (e.g., groups of homogeneous subjects) could be identified (Muthén, 2001; Muthén and Muthén, 2021). Five criteria were used to decide the final number of classes: (a) the Bayesian information criterion (BIC) and (b) the Akaike information criterion (AIC), according to which the model with the smallest value is considered the best model; (c) the Vuong-Lo-Mendell-Rubin (VLMR) test of fit, which compares solutions with different numbers of profiles (a low value of *p* indicates that the *k* model has to be rejected in favor of a model with at least *k*+1 profiles); (d) entropy values, which determine classification quality (values close to 1 indicate clear classification; Celeux and Soromenho, 1996); and (e) the clarity and interpretation of the profiles.

**Figure 1:**
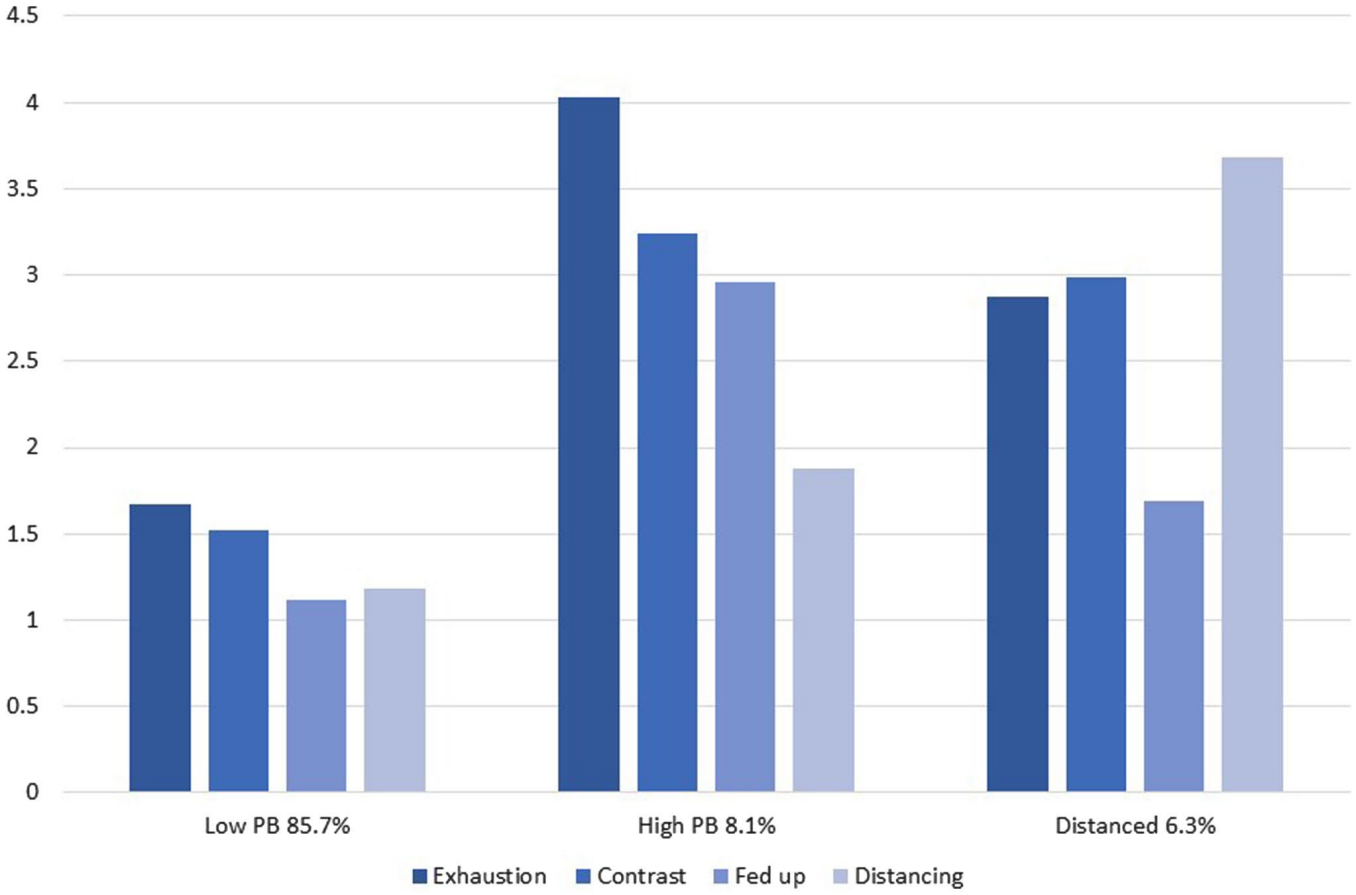
Latent profiles of parental burnout.

Table 2 shows the different fit indices for the compared latent profile solutions. Comparison of the fit indices and profile frequencies showed that when a third group was included in the analyses, the BIC, aBIC, and AIC slightly decreased and entropy value slightly increased compared to the two profile solution, and the profile sizes were acceptable. Also the VLMR test suggested that including a third profile would better fit the data. Thus, because the three profile solution was theoretically meaningful and the goodness-of-fit indices indicated that the third latent group was necessary, the three-latent-group solution was considered the best model. The final three profile solution is presented in Figure 1.

**Table 2:** Fit indices for the compared latent profiles.

Number of profiles	BIC	aBIC	AIC	Entropy	VLMR	Difference in the number of parameters	Value of *p*	Latent class proportio*n* %
1.	13784.77	13759.35	13743.32	–	–	–		
2.	11757.69	11725.92	11705.89	0.96	−7083.16	4	0.01	85/15
3.	11496.68	11439.50	11403.43	0.97	−5977.94	5	0.03	86/6/8
4.	11008.11	10935.05	10888.95	0.97	−5683.71	5	0.20	81/11/4/4

BIC, Bayes information criteria; aBIC, Adjusted Bayes information criteria; AIC, Akaike information criteria; and VLMR, Vuong-Lo-Mendell-Rubin.

The first latent profile (85.7% of the parents) was characterized by a low level of all parental burnout components (Figure 1). The second latent profile (8% of the parents) was characterized by a relatively high parental exhaustion, contrast with previous parental self, and feelings of fed up, but a low level of emotional distancing. The third latent profile (6.3% of the parents) was characterized by an average level of parental exhaustion, contrast with previous self, and feelings of fed up, and a high level of emotional distancing from one’s children. The latent profiles were labeled as *low parental burnout (*e.g., *low PB), high parental burnout (*e.g., *high PB),* and *emotionally distanced* profiles.

Next, to investigate the role of covariates in predicting the three latent profiles of parental burnout, parents’ perceptions of their children’s strengths and difficulties (prosocial skills, hyperactivity, somatic problems, conduct problems, and peer problems) and parents’ gender and educational level were added in the final model separately as covariates using the three-step procedure (Asparouhov and Muthén, 2014). The results for the covariates showed that parents who perceived their children as having high prosocial skills, and who emphasized growth mindset, were more likely to belong to the low PB profile than to the distanced profile (Table 3). Moreover, parents who perceived their children were hyperactive or had problems with peers, more often belonged to the distanced or high PB profiles rather than to the low PB profile. Parents who perceived their children had somatic or conduct problems more often belonged to the distanced or high PB profiles rather than to the low PB profile or to the high PB rather than to the distanced profile. Further, mothers more often belonged to the high PB rather than to the distanced profile, whereas fathers more of the belonged to the distanced rather than to the low PB profile (Table 3). Parents with higher educational level rather belonged to the low PB than to the high PB profile. Further, parents with higher educational level more often belonged to the distanced rather than to the low PB profile.

**Table 3:** Antecedents of parental burnout profiles.

	Distanced vs. low PB	High PB vs. low PB	High PB vs. distanced
SDQ prosocial	−1.06^***^	0.31	−0.31
SDQ hyperactive	1.18^***^	1.49^***^	0.31
SDQ somatic	0.91^**^	1.55^***^	0.64^*^
SDQ conduct problems	1.44^***^	2.54^***^	1.10^*^
SDQ peer problems	1.25^***^	1.55^***^	0.30
Growth mindset	−0.31^***^	−0.15	0.16
Gender	0.53^*^	−0.48	−1.01^*^
Educational level	−0.51^*^	0.26	0.78^*^

*** *p*<0.001;

** *p*<0.01;

* *p*<0.05.

## Discussion

The present study was one of the first person-oriented studies which examined parental burnout profiles using sophisticated statistical methods, i.e., LPA. Parental burnout profiles were examined during an unprecedented time of global COVID-19 pandemic, when in many countries, new regulations and lockdowns took place. The pandemic caused changes in almost every aspect of life, causing severe psychological, social, and financial strain for many families. Due to the pandemic, social contacts have reduced, and the availability of social support and help in child rearing have decreased, which might have increased risk for parental burnout. Because of the pandemic, parents had to balance between work and taking care of their children, and they might have to extent their work beyond regular working hours. Parents have also had fewer places to go for their own leisure activities which typically help in creating a better work and family life balance. All these strains might have increased parental stress, concern about their children, and parental burnout. The present study examined associations between parents’ child-related perceptions and parental burnout profiles among Finnish parents.

### Latent Profiles of Parental Burnout

Although the structure of parental burnout dimensions has been examined to some extent (for examples, see Roskam et al., 2018; Aunola et al., 2020), only two studies so far have examined parental burnout profiles using cluster analysis (Lebert-Charron et al., 2021) or LPA (Hansotte et al., 2021). To the authors’ knowledge, the present study was the first examining the latent profiles of parental burnout measured with the PBA using LPA. The advantage of LPA over traditional cluster analysis is that it provides fit indices for different latent profile solutions, which helps in determining the best fitting final solution for the data. Three latent profiles of parental burnout were identified among Finnish parents, namely, high PB, low PB, and distanced profiles. The largest profile was the low PB profile to which 85.7% of the parents belonged. It was characterized by a low level of all components of parental burnout (e.g., parental exhaustion, contrast with previous parental self, feelings of being fed up, and emotional distancing). The second largest profile was the high PB profile to which 8% of the parents belonged, and it was characterized by a relatively high parental exhaustion, contrast with previous parental self, and feelings of fed up, but a low level of emotional distancing. The third latent profile (6.3% of the parents) was called distanced profile, as it was characterized by a high level of emotional distancing from one’s children, and an average level of parental exhaustion, contrast with previous self, and feelings of fed up. These results are partially in line with previous research showing that approximately 67% of French mothers reported absent or low scores, and 22% reported high scores on parental burnout (Lebert-Charron et al., 2021). In our study, 8% of the parents reported high parental burnout. These differences may be due to also fathers participated in the present study. Previously, it has been find that mothers often report higher parental burnout (Aunola et al., 2020; Furutani et al., 2020; Roskam and Mikolajczak, 2020).

In the present study, emotionally distanced parents separated out as their own profile. These results suggest that small populations (6%) of exhausted parents use emotional distancing as their “reserve” to escape otherwise overwhelming tasks of parenting during the pandemic (see also Cullati et al., 2018). Similarly, Hansotte et al. (2021) found in their study one small (8%) profile in which parents reported high emotional exhaustion and distancing. The results further showed that especially fathers belonged to the emotionally distanced profile. Fathers often spend more time at work than mothers (Nomaguchi et al., 2005), which might make them prone to emotional distancing from their families. Previous studies have also suggested that profiles of emotionally distanced parents are composed of a very specific group of parents who might suffer from other mental disorders (Lebert-Charron et al., 2021). Future studies should examine the characteristics of this profile further.

Importantly, the results showed that different profiles of parental burnout can be identified. Similar to previous studies which have shown that smaller proportions of parents (approximately 20% of mothers) suffer from parental burnout (Séjourné et al., 2018), the results indicated that 14.3% of the parents reported parental burnout symptoms. However, different from most previous studies, 8% of the parents reported high levels of all four burnout symptoms, whereas 6.3% of the parents reported high emotional distancing and altered levels of exhaustion, contrast with previous self, and feelings of fed up. These are important results which show that parental burnout symptoms can manifest in multiple ways among parents. Further interventions to prevent and treat parental burnout could be designed based on these results. For example, parents who suffer from overall high parental burnout may benefit more from directive treatment interventions which target the discrepancy between parenting demands and resources, whereas emotionally distanced parents may benefit from nondirective treatment interventions focusing on active listening, encouragement, and feelings of worth and ability (see also Brianda et al., 2020). However, when compared to the previous studies (Séjourné et al., 2018; Lebert-Charron et al., 2021), in the present study the percentages of parents suffering from burnout during the COVID-19 pandemic were not higher. However, the above mentioned studies targeted only mothers’ experiences, among who parental burnout is often higher (Aunola et al., 2020; Furutani et al., 2020), which may partly explain the results.

### Associations Between Parental Burnout and Parental Beliefs and Perceptions

The role of parental perceptions concerning children’s strengths and difficulties, and growth mindset in determining parents’ belonging to one of the three parental burnout profiles was examined. The results clearly indicated that parents’ concerns about their children’s difficulties (e.g., hyperactivity, somatic, conduct, and peer problems) were associated with parents belonging to one of the burnout profiles, whereas parents whose children had high prosocial skills (e.g., strength) or who emphasized growth mindset were more likely to experience low parental burnout. Previous research examining self-, parent-, and teacher-reports of strengths and difficulties questionnaire has proven its validity as a tool for identifying emotional and behavioral problems in children and adolescents (Tobia and Marzocchi, 2018; Theunissen et al., 2019). Thus, it is possible that parents’ concerns about their children’s difficulties were accurate and manifested as increases in parental burnout. Similarly, previously it has been found that parents whose children have externalized disorders, such as conduct disorders or antisocial behavior, often experience higher levels of parental burnout (Sorkkila and Aunola, 2020) and disengage emotionally from their children (Roskam et al., 2018). Due to the ongoing pandemic, most parents were spending more time at home with their children, and parents were also able to observe their children’s learning and possible related problems much more frequently than before, which might have increased parents’ concerns. Also other fears, worries, and life changes related to the COVID-19 pandemic might have triggered more child-related concerns among parents. On a positive note, children’s strengths and parents’ growth mindset manifested as low parental burnout. Beliefs about mindsets can shape parents’ experiences and attitudes toward their children’s learning (see also Dweck and Yeager, 2019). Parents who employ growth mindset, believe their children’s capacities can be developed over time (Dweck and Yeager), and are often more involved in their children’s education and engage with their children in more constructive ways than parents who employ fixed mindsets (Mueller et al., 2017), which may also reduce parental stress. Growth mindset can reduce stress and enhance resilience among university students (Mosanya, 2020). Similarly, among parents, growth mindset might act as a sign of resilience and protect parents from parental burnout. In future studies, it would be possible to design growth mindset interventions to help reducing parental burnout (see also Rowe and Leech, 2019). Moreover, mothers more often belonged to the high PB rather than to the distanced profile, whereas fathers more of the belonged to the distanced rather than to the low PB profile. These results partly align with findings of some previous studies showing that mothers often score higher in the global parental burnout scale (Aunola et al., 2020). Contrary some previous findings showing that educational level is not associated with parental burnout (Mikolajczak et al., 2018; Roskam et al., 2018; Aunola et al., 2020), the present results showed that parents with higher educational level suffered less from parental burnout. One reason for these differences in the findings may be methodological differences, i.e., the present study being person-oriented compared to previous variable-oriented studies.

### Limitations

This study has some limitations which should be taken into account when generalizing the findings of the present study. First, the study design was cross-sectional, which made it not possible to examine the order of the associations (e.g., whether parental perceptions predict parental burnout profiles or *vice versa*). Similar associations should be examined in future studies using longitudinal designs. Second, the parental burnout assessment used in the present study was a shortened version of the original 23-item PBA scale (Roskam et al., 2018) which may have affected the results. More studies would be needed examining latent profiles of parental burnout using the original PBA scale. Third, some variables used in the present study showed only moderate reliability, and even values between 0.50 and 0.70 are considered as moderate reliability (Perry et al., 2004), more studies in the future would be needed to explore similar constructs further. Fourth, other variables which were not examined in the present study might have affected the results. For example, parental burnout is often associated with depressive symptoms, lower self-esteem, and sleep disruptions (Lindström et al., 2011; Mikolajczak et al., 2019; Aunola et al., 2020). More studies would be needed in the future to examine whether these variables are associated with latent profiles of parental burnout.

## Conclusion

The present study showed importantly that by using person-oriented research (LPA), it is possible to identify distinct homogenous profiles of parents who suffer from parental burnout. The results indicated that most parents (85.7%) typically show low parental burnout, whereas smaller percentages of parents belong to high (8%) or distanced (6.3%) parental burnout profiles. Similarly, previous studies have shown that the majority of parents (between 60 to 80%) typically reports low or nonexistent parental burnout symptoms (Roskam et al., 2018; Séjourné et al., 2018; Lebert-Charron et al., 2021). Unlike burned out employees, burned out parents cannot take sick leave or take extended breaks from parenting (Mikolajczak et al., 2019). The COVID-19 pandemic also decreased normal social interactions and families are spending more time among themselves. Having no escape from parenting may prompt some burned out parents to emotionally distance from their children, as our results showed. Worries and fears about the virus spreading, and other social, psychological, and financial strains that the pandemic caused might have increased parents’ concerns about their children’s behavioral and emotional difficulties. In future studies and intervention designs, it would be important to take into account the type of parental burnout profiles each parent belongs to. For example, some parents might benefit from reducing the discrepancy between parenting-related demands and resources, whereas other parents may benefit more from active listening and encouragement (see also Brianda et al., 2020). Moreover, the present study examined antecedents of parental burnout profiles, using a shortened version of PBA. Recently, the outcomes of parental burnout (measured with the original PBI) profiles were examined, showing different profiles of parental burnout were associated with different consequences in terms of neglect and violence toward children (Hansotte et al., 2021). More studies would be needed to examine the outcomes of parental burnout profiles using the PBA scale. In addition, the notion that some parents suffer from parental burnout is relatively new, and more information and public discussion concerning the topic would be needed. Increasing discussion about parental burnout and related factors would help parents to better understand their symptoms and, if necessary, seek for help. Moreover, brief instruments could be develop to be used at places, such as maternity clinics and healthcare centers, to screen and prevent possible symptoms of parental burnout, and to identify possible risk groups of parents prone to such symptoms (see also Aunola et al., 2020). Such screenings could be conducted, for example, in regular intervals at the same time when parents take children to their regular checkups in order to enhance families wellbeing.

## Data Availability Statement

The original contributions presented in the study are included in the article/supplementary material, further inquiries can be directed to the corresponding author.

## Ethics Statement

The studies involving human participants were reviewed and approved by the University of Helsinki Ethical Review Board. The patients/participants provided their written informed consent to participate in this study.

## Author Contributions

KU contributed to the writing of the manuscript and performed the statistical analyses. KS-A contributed to the design of the study and writing of the manuscript. All authors contributed to the article and approved the submitted version.

## Conflict of Interest

The authors declare that the research was conducted in the absence of any commercial or financial relationships that could be construed as a potential conflict of interest.

## Publisher’s Note

All claims expressed in this article are solely those of the authors and do not necessarily represent those of their affiliated organizations, or those of the publisher, the editors and the reviewers. Any product that may be evaluated in this article, or claim that may be made by its manufacturer, is not guaranteed or endorsed by the publisher.
